# The Influence of Early Temperament on Language Development: The Moderating Role of Maternal Input

**DOI:** 10.3389/fpsyg.2018.01527

**Published:** 2018-08-28

**Authors:** Maria Spinelli, Mirco Fasolo, Prachi E. Shah, Giuliana Genovese, Tiziana Aureli

**Affiliations:** ^1^Department of Neuroscience, Imaging and Clinical Sciences, University “G. d’Annunzio” Chieti - Pescara, Chieti, Italy; ^2^Department of Pediatrics, University of Michigan, Ann Arbor, MI, United States; ^3^Department of Psychology, University of Milano-Bicocca, Milan, Italy

**Keywords:** temperament, language development, maternal input, infant directed speech, longitudinal study, moderation

## Abstract

Temperament is an individual aspect that strictly affects infants and children engagement with the environment and it is supposed to play a role in the acquiring of new competences. Several studies focused on the possible influence of temperament in the process of language acquisition in early childhood reporting not consistent findings. Since maternal input is a variable that has been widely associated with infant language development this longitudinal study aimed to explore the role of the quality of maternal input in the temperament-language association. We hypothesized that the longitudinal association between early infant temperament and language production is moderated by the quality of maternal input during the first year of life. Infant temperament at 3 months and maternal linguistic input (lexical diversity and syntactic complexity) during spontaneous mother–infant interactions at 6, 9, and 12 months were assessed. Language competences were evaluated at the end of the second year: language production at 18 months with the CDI and child syntactic complexity at 24 months during spontaneous speech. Results showed significant moderating effects of syntactic complexity and lexical variability of maternal input at 6 and 9 months on the association of duration of orienting abilities and later language production. Infants with greater attentional abilities and with mothers who spoke to them with a more complex and variable input showed the better language outcomes. The association between infant distress to limitations and child language was not moderated by maternal input. No effects were found when considering the temperamental scale smile and laugher. Attentional control temperamental characteristics could help the infant to be more focus on maternal input throughout the first year of life and could consequently facilitate language development. Our findings underlined the necessity to explore infant development considering the interaction between individual and contextual factors.

## Introduction

Temperamental traits are biologically based characteristics manifest early in life, that contribute to individual differences in regulating and modulating emotion, attention, behavior, and motor activity (Rothbart, 1981, 2007; Gartstein and Rothbart, 2003). Because development emerges in the context of bidirectional interactions between the infant and his environment (Sameroff, 1975), temperament can influence how the infant receives and responds to stimuli in his environment, which can play a role in subsequent development. The developmental role of temperament has been mostly studied with respect to socio-emotional domain where it has been associated with both externalizing and internalizing problems (i.e., Ullsperger et al., 2016; Rubin et al., 2017) and social competence (i.e., Baer et al., 2015; Penela et al., 2015). Temperament is thought to play a role also in language acquisition, and is believed to partially account for the variability in the rate and style of language acquisition (e.g., the age of the first word, and rate of vocabulary development and syntactic emergence) during the first 2 years of life (Bates et al., 1991; Lieven, 1997). Nevertheless, research in this domain rather focused on the caregiver contributions, with a rich body of literature analyzing the quality of linguistic maternal input directed to the child and finding consistent results on its impact on infant linguistic outcomes (e.g., the quality of linguistic maternal input directed to the child; see Soderstrom, 2007, for a review). Less studies exist, on the child’s contribution such as that provided by the influence of temperamental traits, with the few existing studies reporting inconsistent findings (e.g., Dixon and Smith, 2000;Wolfe and Bell, 2007; Kubicek and Emde, 2012). One potential explanation for these discrepant results is the failure to consider a transactional approach (Sameroff, 1975), which includes both the child’s contributions to language development (i.e., temperament) and the caregiver’s role (e.g., quality of the maternal input). This study aims to address this gap in the literature by exploring the interaction between these two variables as factors of influence on early language acquisition.

### Temperament and Language

The first year of life is a period characterized by the rapid emergence of semantic and syntactic skills, which allows infants to decode the streams of speech directed to them and begin to associate sounds with symbolic meaning. Two temperamentally based characteristics, mainly an infants’ attentional control and the capacity for self-regulation, are thought to play a role in facilitating or inhibiting this process (Canfield and Saudino, 2016), although research exploring this association has yielded discrepant results.

Regarding the temperamental characteristic of attention control, several studies have demonstrated a positive association between temperament and language. Infants who demonstrated better attentional abilities, manifest by higher scores in duration of orienting and persistence at 7 and 13 months, also demonstrated higher language comprehension at the end of the first year (Dixon and Smith, 2000; Morales et al., 2000), and greater language vocabulary productivity at 21 months (Dixon and Shore, 1997; Dixon and Smith, 2000; Salley et al., 2013). These findings suggested that more optimal attentional capacities (e.g., greater sustained attention to the external environment) might facilitate a child’s abilities to focus on linguistically relevant events thereby contributing to vocabulary development. Other research yielded contradictory findings, with one study (c.f. Wolfe and Bell, 2007) demonstrating a negative association between the duration of orienting at 8 months of age and receptive vocabulary at 4.5 years, and other studies finding no association between attentional control temperament and language development (Kubicek and Emde, 2012; Pérez-Pereira et al., 2016). These disparate findings suggest a need for additional research to further examine the association between the temperamental trait of attention control and language development.

In addition to attention regulation, there is some research suggesting that individual differences in emotion expression and regulation are associated with language development (Pérez-Pereira et al., 2016). Several studies have found an association between higher positive affect (e.g., higher smiling, laugher, and easier soothability) at 7 and 10 months, and better language comprehension at 12 months of age (Morales et al., 2000) and better expressive language at 14 months (Laake and Bridgett, 2014). Similarly, high levels of affect–extraversion at 2 years of age have also been associated with advanced receptive and expressive language at age 3, and better receptive language skills later at age 7 (Slomkowski et al., 1992). This association appears to be bidirectional, wherein children with more advanced language development at 13 months (i.e., “early talkers”) were also more likely to express greater positive affect and lower negative affect at 15 months (Kubicek and Emde, 2012). Taken together, this literature suggests that a greater expression of positive affect may help foster greater social exchanges in infancy during a critical period of language development which may help facilitate language development in the infant and toddler period.

There is also a body of literature which suggests that more negative affect (e.g., difficult temperament) is adversely associated with language development. McNally and Quigley (2014) found that infants rated as having more difficult temperament at 9 months had lower global language scores at 3 years of age, with similar associations demonstrated for infants at 21 months of age (Dixon and Smith, 2000; Salley and Dixon, 2007). One potential mechanism explaining this association is that children’s difficult temperament may interfere the utilization of attentional resources needed to process linguistically relevant information and may thus be associated with suboptimal language development during a critical period of language acquisition.

However, despite a body of literature suggesting an association between emotion regulation and language development, there is also a body of literature which has demonstrated contradictory findings. Some studies have failed to find association between the negative affect in infancy (e.g., distress to limitations scale and difficult temperament) and language competencies in the toddler years (Dixon and Smith, 2000; Morales et al., 2000; Westerlund and Lagerberg, 2008; Canfield and Saudino, 2016). However, adding to the complexity of our understanding of the association between emotion regulation and language, research by Moreno and Robinson (2005) found that both greater expressions of joy and greater expressions of anger at 8 months were associated with better expressive language at 30 months, suggesting that emotional expression (both positive and negative) may play a role in facilitating language development by providing opportunities for the child to develop language through dyadic exchanges (Molfese et al., 2010).

An alternate view has suggested that it is neither positive or negative affective states which are associated with more optimal language development, but rather neutral affective states. Bloom (1990) proposed that more time spent in neutral states might facilitate early language learning by allowing for the reflective stance needed to construct the mental meanings for learning words. As a support of this proposal, Bloom and Capatides (1987) and Bloom et al. (1988, 2001) studies indicated a detrimental influence of both negative and positive affect on language acquisition, while more neutral affect was associated with earlier world learning.

Taken together, these disparate findings suggest that while some research suggests that attentional and emotional temperamental aspects may play a role in fostering interactions which can enhance language development, further research is needed to better understand the mechanism of association between attention, emotion regulation, and the development of language competencies.

### Maternal Input, Temperament and Language Development

One potential explanation for the disparate results in the cited literature is the lack of consideration of other aspects that may be associated with language acquisition, and which may moderate the association between temperamental characteristics and language competence, such as the quality of maternal linguistic input (e.g., Soderstrom, 2007; Saint-Georges et al., 2013; Golinkoff et al., 2015). Research found that the variation in the amount, richness and structure of talk addressed to prelinguistic children revealed to be promotional for subsequent linguistic development (e.g., Huttenlocher et al., 1991; Hampson and Nelson, 1993; Rowe, 2008; Weisleder and Fernald, 2013; Newman et al., 2016), with infants who are exposed to more variable and complex speech have greater opportunities to foster skills related to language interpretation and speech segmentation. In addition, greater maternal lexical and syntactic variety during the first year of infant life is associated with more optimal child language competence in the second year of life (Huttenlocher et al., 1991; Bornstein et al., 1998; Goodman et al., 2008).

Since the demonstrated relevance of maternal input and the discrepancy in research on temperament, our aim was to find whether the quality of the maternal linguistic input would act as a moderating factor for the association between infant temperament and infant language development (Conture et al., 2013). We theorize that a child’s temperamental characteristics may play a role in subsequent language development by affecting the way the infant receives and responds to input from the linguistic environment, and that the quality of the maternal linguistic input may moderate the association between infant temperament and infant language development. To the best of our knowledge, only two studies have examined the topic in this way. Karrass and Braungart-Rieker (2003) reported that at 12 months maternal responsiveness to their children (e.g., affective warmth) moderated the association between the child’s temperamental characteristic of “distress to novelty” and language development 4 months later, with infants who demonstrated lower distress to novelty manifesting better language abilities in the context of more responsive mothering. In addition, Laake and Bridgett (2018) showed that for infants with more positive affect at 10 months, more supportive maternal interactions were associated with better expressive language at 14 months. While these studies highlight the interactive effects of infant temperament and responsive caregiving with more optimal language development, neither study examined the role of the maternal linguistic environment as a potential moderator of the association between temperament and language development.

### The Present Study

Most studies on temperament and language development focus on children from 24 to 36 months of age. However, if the role of temperament on infants’ responses to maternal linguistic stimuli is to be examined, it is necessary to examine these processes from an earlier age. We may expect infant temperament during the first year of life to play a role in language acquisition, whereas temperament in preschool age may manifest in the child’s response to social contexts, potentially manifest as shyness, social inhibition and reticence to talk with others. While many studies have demonstrated correlations between these variables, current research has failed to identify the mechanisms underlying these associations, suggesting the need for longitudinal studies to examine the processes by which temperament contributes to language development in infancy and toddler years.

In this study, we focused on three dimensions of temperament (infant attention, positive affect, and negative affect), using validated scales from the Infant Behavior Questionnaire (Gartstein and Rothbart, 2003), that have been previously associated with language development. Infant attention was assessed by the duration of orienting scale, positive affect was assessed by the smile and laughter scale, and negative affect was assessed by the distress to limitations scale. Building on previous research (c.f. Saffran et al., 2006), the influence of temperament on infant language has been measured with respect to productive lexicon at 18 months, and syntactic competence at 24 months of age, whereas the quality of maternal input was measured in term of lexical variability and syntactic complexity. We hypothesized that children with temperamental skills characterized by higher attention and more positive, and less negative affect (e.g., higher duration of orienting, higher smile and laugher and lower distress to limitations) would have better linguistic skills in the context of greater quality of maternal input, than children who were lower in those skills.

## Materials and Methods

### Participants

Seventy mother–infant dyads participated at the study. Participants were drawn from a larger longitudinal study on mother–infant relationship (Coppola et al., 2016) and were recruited from the public hospital of an urban area within 2 days of the baby’s birth. Mothers who signed an informed consent both for their own participation in the research as well as for the children’s participation were included in the study. Mothers and infants attended the Lab of the University when the infant was 3, 6, 9, 12, 18, and 24 months old.

Inclusion criteria were as follows: infants were born full-term, belonged to bi-parental Italian families, had mothers identified as the primary caregiver, mothers’ age >21 years. Infants were excluded if they had medical complications at birth, had experienced hospitalizations or had been diagnosed for medical or psychological delays/disorders. The study has been reviewed and approved by the Ethical Committees of the University G. D’Annunzio Chieti-Pescara.

### Procedure

At 3 months, mothers completed the Infant Behavior Questionnaire-Revised (IBQ-R; Gartstein and Rothbart, 2003). At 6, 9, 12, and 24 months, mother and infants were videotaped when interacting in a 3-min face-to-face session. A trained researcher transcribed in CHAT format (MacWhinney, 2000) each verbal utterance produced by mothers directed to the child (during the 6, 9, and 12 months sessions). An utterance was defined as any sequence of maternal verbal production delimited by an auditory pause (500 ms or more of non-speech) or defined as an understandable change in the conversational turn (Kitamura and Burnham, 2003).

At the 24-month time visit, each child verbal utterance was transcribed, excluding the utterances that were characterized as crying, physiological vocalizations, or discomfort sounds. Children’s utterances were counted as a single unit of transcription when separated by at least 1 s. Following Vihman and McCune (1994) a verbal utterance was identified as a word when: it was phonetically similar to the adult word; it was treated as meaningful by the mother; the child uses it in multiple and appropriate contexts.

Inter rater reliability was achieved by having a second coder transcribing independently 20% of videotapes. Excellent inter-rater reliability was obtained for all assessments of mother and child vocalizations: child’s utterances: κ = 94%; child’s verbal productions: κ = 99%; mother’s utterances: κ = 98%; mother’s verbal productions: κ = 99%. The coders were blind to study hypothesis.

When children were 18 months of age mothers also completed the Italian version of the McArthur-Bates CDI (Caselli and Casadio, 1995).

### Measures

#### Infant Temperament

As no official Italian version of the IBQ-R (Gartstein and Rothbart, 2003) was available at the time we began the study, we created our own version, which was derived from a translation and back translation of the original form (Aureli et al., 2015). After validating the full form, we created a shorter form to make the instrument less demanding for mothers. A new validation process was then undertaken, which produced an instrument with 103 items and 14 scales, the same as in the full version (activity level, distress to limitations, fear, duration of orienting, smile and laughter, high intensity pleasure, low intensity pleasure, soothability, falling reactivity/rate of recovery from distress, cuddliness, perceptual sensitivity, sadness, approach, and vocal reactivity).

For this study, we used three subscales: Duration of orienting (to assess infant attention), smile and laughter (to assess positive affect), and the distress to limitations scale (to assess negative affect). The mean scores in the duration of orienting (attention to and/or interaction with a single object for extended periods of time), smile and laughter (smiling or laughter during general caretaking and play), and distress to limitations (fussing, crying, or showing distress while in a confining place; or position or in caretaking activities; or unable to perform a desired action) were calculated (Cronbach’s *α*: Duration of orienting: 0.89; smile and laughter: 0.82; distress to limitations: 0.87).

#### Maternal Verbal Input

The measures of lexical and syntactic characteristics of maternal input were calculated on all the utterances produced by mothers during the mother–child interaction at 6, 9, and 12 months:

- Lexical variability was measured by the frequency of word types, i.e., the number of different word roots, per minute. It was calculated using the MOR command of CHAT program (MacWhinney, 2000).
- Syntactic complexity was measured by computing the mean length of utterance (MLU), i.e., the ratio of words to utterance, using the MLU command of CHAT program (MacWhinney, 2000).

#### Child Language Competences

##### Vocabulary production competence

At 18 months, mothers completed the Italian version of the McArthur-Bates CDI (Caselli and Casadio, 1995). The Italian version of CDI is modeled after the English version in terms of overall format, number and type of lexical categories and number of items. It has been validated and it is widely used in studies on language development (e.g., Fasolo et al., 2010; Zampini et al., 2012). The CDI consists of a vocabulary list of 408 words, for which both comprehension and production is assessed. Vocabulary production was evaluated at 18 months assessed by the total number of words identified from the CDI. Two coders counted the number of words identified by the mothers.

##### Syntactic competence

At 24 months a direct measure of child syntactic complexity was evaluated through the analysis of spontaneous speech produced during the mother–infant interactions and the calculation of the MLU, i.e., the ratio of words to utterance, using the MLU command of the CHAT program (MacWhinney, 2000).

#### Analytic Plan

The associations among infant temperament, maternal input, and child language skills were first evaluated using correlational analyses.

To examine the main and interactive effects of temperamental characteristics and maternal input and its effect on language abilities, we tested a series of moderation models using the SPSS PROCESS macro (Hayes, 2017). Moderation tests the likelihood that the effect of an independent variable on a dependent variable is affected by values of a second moderating variable. Five thousand bootstrap resamples were used to generate 95% confidence intervals to estimate the size and significance of the effects. All studied variables were standardized before testing moderation models to allow for the comparison of the effects. Following Hayes (2017) the moderating effect was demonstrated by evidence of a significant interaction (*p* < 0.05) of the independent variable and the moderator. Finally, to facilitate interpretation of significant moderations we plotted conditional effects (simple slopes) for low (16th percentile), medium (50th percentile), and high (84th percentile) levels of the moderating variables (maternal input lexical variability and syntactic complexity).

To check for multicollinearity we calculated the VIFs by computing regression analysis with all the data. The VIFs were between 1.02 and 2.01 showing a low risk for multicollinearity in our models. From a direct *post hoc* estimation, the power (1-β err prob) to find an effect size of interest (*f*^2^ = 0.15), with a probability level alpha = 0.05, and with 6 predictors, was 0.84 for a sample size of 61.

## Results

### Descriptive Statistics

The mothers’ mean age was years 34 (*SD* = 4.69; range = 20–44), the average number of years of education was 15 (*SD* = 3.02; range = 8–18), and 74% (*n* = 51) of them were employed. For the infants, 49% were male (*n* = 34) and 51% (*n* = 35) were firstborns. Seven mothers did not complete the CDI at 18 months, and six children did not participate at the 24 months session. Because there were no differences between these dyads and the others with complete data on measures of infant temperament and maternal language data (all *p*’s > 0.05 at *t*-tests), these mother–infant dyads were excluded from the corresponding models but included in the models were the data were available. The descriptive statistics for infant temperament, infant language competences at 15, 18, and 24 months and maternal input at 6, 9, and 12 months are delineated in **Table 1**.

**Table 1:** Descriptive statistics of study’s variables.

	Full sample		Boys		Girls				
	*M*	*SD*	*M*	*SD*	*M*	*SD*	*T*	*df*	*p*
**Infant temperament**									
IBQ 4 m duration of orienting	4.01	1.00	4.11	1.07	3.90	0.93	0.85	67	0.39
IBQ 4 m smile and laughter	4.89	0.93	4.92	1.09	4.87	0.76	0.25	68	0.80
IBQ 4 m distress to limitations	3.35	0.65	3.44	0.62	3.25	0.67	1.17	68	0.25
**Maternal input**									
Types per minute 6 months	11.83	3.39	11.83	3.53	11.83	3.29	0.00	68	0.99
Types per minute 9 months	10.56	2.51	10.28	2.41	10.83	2.61	-0.91	68	0.36
Types per minute 12 months	12.77	2.66	12.47	3.02	13.06	2.25	-0.93	63	0.36
MLU 6 months	2.70	0.41	2.70	0.44	2.70	0.39	0.06	68	0.95
MLU 9 months	2.48	0.39	2.50	0.39	2.46	0.39	0.38	68	0.71
MLU 12 months	2.78	0.39	2.72	0.40	2.83	0.38	-1.23	68	0.23
**Child language competences**									
CDI production 18 months	67.88	75.90	225.30	91.42	267.59	87.61	-1.80	54	0.07
MLU 24 months	1.38	0.37	1.23	0.22	1.50	0.44	-3.03	62	<0.01**

^∗^p < 0.05, ^∗∗^p < 0.01.

Infant temperamental characteristics and maternal input did not differ by gender, however, girls showed more syntactic complexity at 24 months. Maternal educational level was not associated with any of the infant and maternal variables (all *p*’s < 0.05), and as a result, this variable was not included in our multivariate models.

### Correlational Analyses

We found significant correlations among infant temperament, maternal input and child language competencies, with results displayed in **Table 2**.

**Table 2:** Correlational analyses.

	1	2	3	4	5	6	7	8	9	10	11
**Infant temperament**					
1 IBQ 4 m duration of orienting	–										
2 IBQ 4 m smile and laughter	0.14	–									
3 IBQ 4 m distress to limitations	-0.20	-0.24*	–								
**Maternal input**					
4 Types per minute 6 months	0.09	-0.05	-0.14	–							
5 Types per minute 9 months	0.10	0.16	-0.10	0.63**	–						
6 Types per minute 12 months	0.13	0.17	-0.19	0.60**	0.64**	–					
7 MLU 6 months	0.05	-0.04	0.02	0.51**	0.49**	0.40**	–				
8 MLU 9 months	0.23	-0.07	0.03	0.42**	0.54**	0.41**	0.67**	–			
9 MLU 12 months	0.20	0.16	-0.26*	0.34**	0.38**	0.49**	0.53**	0.53**	–		
**Child language competence**					
10 CDI production 18 months	0.29*	0.12	0.20	0.08	0.10	0.13	-0.04	0.16	0.16	–	
11 MLU 24 months	0.30*	0.21	-0.16	0.00	0.21	0.10	-0.15	0.19	0.14	0.53**	–

^∗^p < 0.05, ^∗∗^*p* < 0.01.

Measures of maternal input were highly correlated over time, with maternal quality and MLU demonstrating a positive correlation both at the same age and among the different ages. This indicated that, even if maternal input varies over time, the speech of each mother maintains a similar linguistic quality. Regarding infant temperament, domains of temperament were highly correlated with each other, with higher scores in the IBQ smile and laughter scale associated with lower scores in the IBQ distress to limitations scale. There were several significant and positive associations among child language scores. Children with higher CDI production scores at 18 months showed a higher MLU at 24 months.

There was no association between infant temperament and maternal input variables, with the exception of maternal MLU at 12 months. Only one domain of infant temperament was correlated with infant language acquisition. Infants who were rated higher on the IBQ duration of orienting scale showed higher vocabulary production at 18 months and more syntactic variability at 24 months. The quality of maternal input at 6, 9, and 12 months was not associated with any of the language competence variables.

### Moderation Analysis

To test whether maternal linguistic input moderated the association between infant temperament characteristics and infant language outcomes, we tested several models one for each IBQ scale as the predictor (IBQ duration of orienting, IBQ smile and laugher, and IBQ distress of limitations) moderated by each maternal input (MLU and types/min at 6, 9, and 12 months), predicting both child language outcomes (CDI production at 18 months and child MLU at 24 months). To improve the precision of the standardized estimates of our multivariate model, maternal input at the other ages and child gender were also included as covariates.

### IBQ Duration of Orienting Effect on Language Competence Moderated by Maternal Input

#### Moderator: Maternal Lexical Variability (i.e., Types Frequency Per Minute)

**FIGURE 1:**
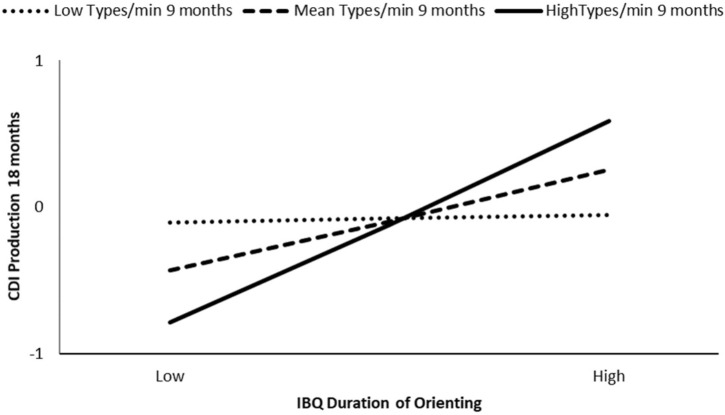
Moderation effect of maternal types/min at 9 months on IBQ duration of orienting effect on CDI production at 18 months.

At 18 months, the association between infant IBQ Duration of orienting and CDI production was not moderated by maternal lexical variability (i.e., types/min) at 6 months (β = 0.13, *p* = 0.28), or maternal lexical variability at 12 months (β = -0.01, *p* = 0.95), however, we did find evidence of moderation by maternal lexical variability at 9 months (β = 0.28, *p* = 0.03). The model at 9 months explained the 20% of the variance, and the interaction was significant at the 50th percentile (β = 0.34, *p* = 0.01) and the 84th percentile (β = 0.69, *p* < 0.01) values of maternal types/min. Higher IBQ duration of orienting competences were associated with higher vocabulary size at 18 months only for infants who experienced mothers with medium and high lexical variety during the interaction at 9 months (see **Figure 1**).

**FIGURE 2:**
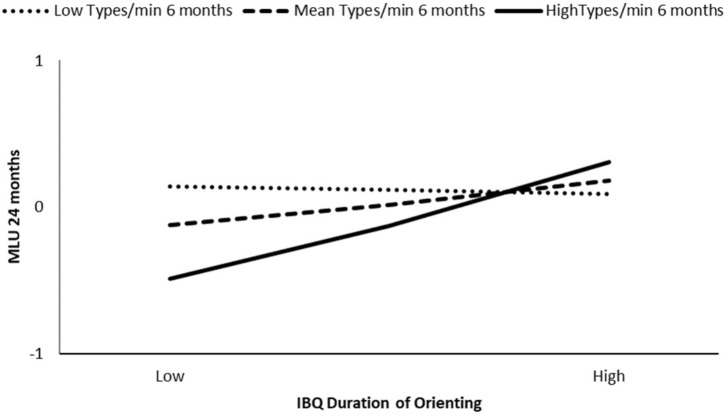
Moderation effect of maternal types/min at 6 months on IBQ duration of orienting effect on child MLU at 24 months.

**FIGURE 3:**
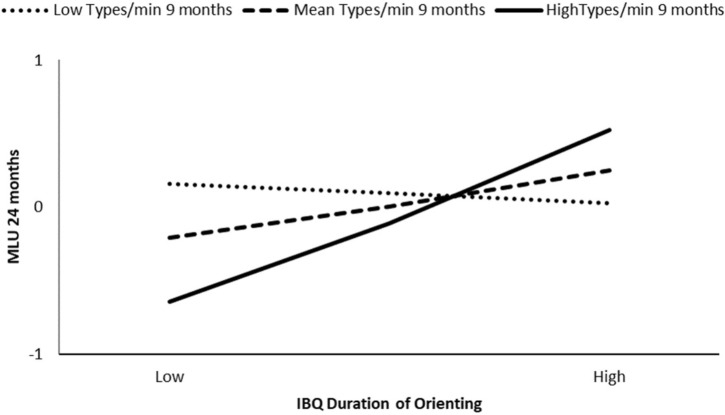
Moderation effect of maternal types/min at 9 months on IBQ duration of orienting effect on child MLU at 24 months.

At 24 months, we found that the association between infant IBQ duration of orienting and child MLU at 24 months was moderated by maternal lexical variability (i.e., types/min) at 6 months (β = 0.27, *p* = 0.04) and 9 months (β = 0.36, *p* < 0.01), but we did not find significant moderation effect of maternal lexical variability at 12 months (β = -0.01, *p* = 0.92). The model at 6 months explained the 26% of the variance, and the interaction was significant for types/min at the 84th percentile (β = 0.46, *p* < 0.01) (see **Figure 2**). The model at 9 months explained the 29% of the variance and the interaction was significant for types/min at the 50th percentile (β = 0.26, *p* = 0.03) and at the 84th percentile (β = 0.67, *p* < 0.01) (see **Figure 3**). Higher IBQ duration of orienting competences were associated with higher child syntactic competence at 24 months for high maternal lexical variability at 6 months, and medium and high maternal lexical variability at 9 months.

#### Moderator: Maternal Syntactic Complexity (i.e., MLU)

**FIGURE 4:**
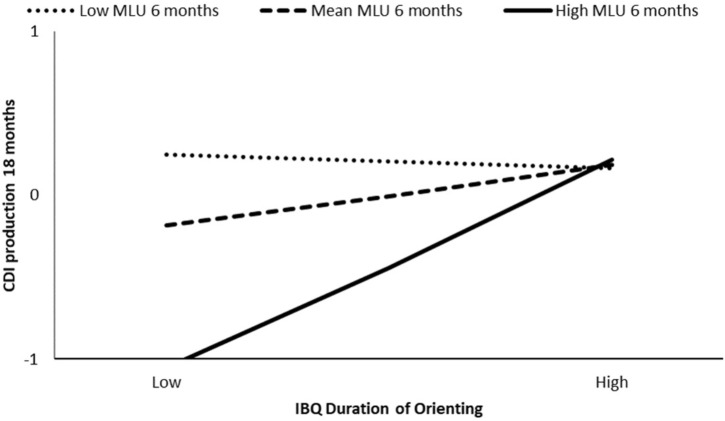
Moderation effect of maternal MLU at 6 months on IBQ duration of orienting effect on CDI production at 18 months.

The association between infant duration of orienting and infant 18 months CDI (number of words) was moderated by maternal MLU at 6 months (β = 0.31, *p* = 0.03), but not by maternal MLU at 9 or 12 months (β = 0.16, *p* = 0.23 and β = 0.01, *p* = 0.97, respectively). The model at 6 months explained the 26% of the variance, and the interaction was significant for MLU at the 84th percentile (β = 0.64, *p* < 0.01). Low IBQ duration of orienting competencies were associated with less language production at 18 months for infants who experienced mothers with high syntactic complexity during the interaction at 6 months (see **Figure 4**).

**FIGURE 5:**
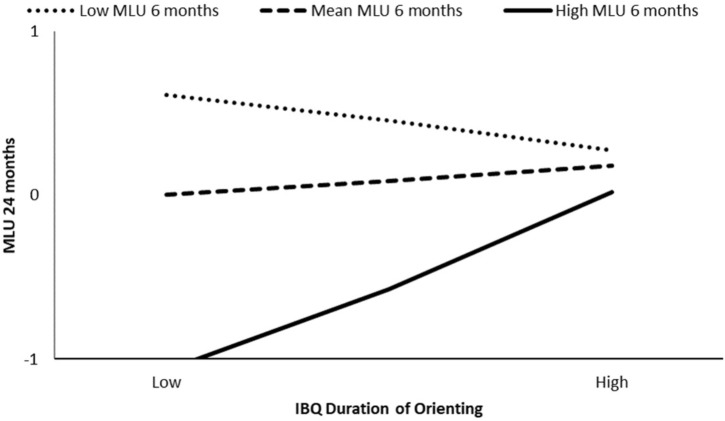
Moderation effect of maternal MLU at 6 months on IBQ duration of orienting effect on child MLU at 24 months.

The association between infant duration of orienting and child MLU at 24 months was moderated by maternal MLU at 6 months (β = 0.37, *p* < 0.01) but was not moderated by maternal MLU at 9 months (β = 0.22, *p* = 0.09) or at 12 months (β = 0.06, *p* = 0.61) (**Table 3**). The model at 6 months explained the 40% of the variance, and the interaction was significant for maternal MLU at the 84th percentile (β = 0.63, *p* < 0.01). Lower IBQ duration of orienting was associated with lower syntactic competence at 24 months for infants whose mothers spoke with higher syntactic complexity at 6 months (see **Figure 5**).

**Table 3:** Moderation results, predictor IBQ Duration of Orienting.

	CDI production 18 m		Child MLU 24 m			CDI production 18 m		Child MLU 24 m	
	*ß*	*SE*	*ß*	*SE*		*ß*	*SE*	*ß*	*SE*
IBQ duration of orienting	0.25	0.14	0.20	0.13	IBQ duration of orienting	0.21	0.13	0.11	0.11
Mother types/minute 6 m	0.03	0.17	-0.11	0.16	Mother MLU 6 m	-0.27	0.17	-0.43**	0.14
IBQ × Types/minute 6 m	0.13	0.12	0.27*	0.13	IBQ × MLU 6 m	0.31*	0.14	0.37**	0.13
Mother types/minute 9 m	0.04	0.18	-0.01	0.16	Mother MLU 9 m	0.20	0.16	0.28	0.15
Mother types/minute 12 m	0.01	0.18	0.09	0.16	Mother MLU 12 m	0.22	0.15	0.30*	0.14
Gender	0.34	0.27	0.75*	0.24	Gender	0.27	0.25	0.57*	0.22
IBQ duration of orienting	0.34*	0.12	0.25*	0.12	IBQ duration of orienting	0.21	0.13	0.11	0.12
Mother types/minute 9 m	0.01	0.17	-0.07	0.16	Mother MLU 9 m	0.22	0.17	0.28	0.15
IBQ × Types/minute 9 m	0.28*	0.13	0.36**	0.13	IBQ × MLU 9 m	0.16	0.13	0.22	0.13
Mother types/minute 6 m	0.02	0.17	-0.10	0.15	Mother MLU 6 m	0.25	0.16	0.31**	0.14
Mother types/minute 12 m	0.03	0.16	0.16	0.15	Mother MLU 12 m	-0.29	0.17	-0.42*	0.15
Gender	0.33	0.26	0.66**	0.24	Gender	0.37	0.25	0.67**	0.23
IBQ duration of orienting	0.30*	0.14	0.26	0.14	IBQ Duration of orienting	0.23	0.14	0.13	0.13
Mother types/minute 12 m	0.03	0.18	0.16	0.17	Mother MLU 12 m	0.22	0.16	0.27	0.15
IBQ × Types/minute 12 m	-0.01	0.11	-0.01	0.11	IBQ × MLU 12 m	0.01	0.13	0.06	0.12
Mother types/minute 6 m	0.04	0.18	-0.05	0.16	Mother MLU 6 m	-0.27	0.18	-0.41*	0.15
Mother types/minute 9 m	0.03	0.18	-0.04	0.17	Mother MLU 9 m	0.23	0.18	0.31	0.16
Gender	0.39	0.27	0.81**	0.26	Gender	0.38	0.26	0.72**	0.24

m, months. ^∗^p < 0.05, ^∗∗^p < 0.01.

### IBQ Smile and Laugher Effect on Language Acquisition Moderated by Maternal Input^1^

#### Moderator: Maternal Lexical Variability (i.e., Types Frequency Per Minute)

The^1^ association between IBQ smile and laughter and CDI production at 18 months, was not moderated by the quality of maternal lexical variability at 6, 9, or 12 months. Similarly, maternal lexical variability at 6, 9, or 12 months did not moderate the association between infant positive affect (i.e., IBQ smile and laughter) and child MLU at 24 months.

#### Moderator: Maternal Syntactic Complexity (i.e., MLU)

None of the models examining the moderating role of maternal MLU between IBQ smile and laugher and CDI production at 18 months and child MLU at 24 months were significant.

### IBQ Distress to Limitations Effect on Language Acquisition Moderated by Maternal Input

#### Moderator: Maternal Lexical Variability (i.e., Types Frequency Per Minute)

None of the moderating effects of maternal lexical variability between IBQ distress to limitations and child CDI at 18 months were significant (see **Table 4**). However, despite no moderation effect, the temperamental characteristic of IBQ distress to limitations had a near-significant main effect in the 6 months (β = 0.28, *p* = 0.05), 9 months (β = 0.27, *p* = 0.06), and 12 months (β = 0.28, *p* = 0.05) models. High IBQ distress to limitations values were independently associated with better language production at 18 months, albeit at a level that trended toward significance.

**Table 4:** Moderation results, predictor IBQ distress to limitations.

	CDI production 18 m		Child MLU 24 m			CDI production 18 m		Child MLU 24 m	
	*ß*	*SE*	*ß*	*SE*		*ß*	*SE*	*ß*	*SE*
IBQ distress to limitations	0.28	0.14	0.12	0.13	IBQ distress to limitations	0.29*	0.14	0.16	0.12
Mother types/minute 6 m	-0.01	0.18	-0.01	0.17	Mother MLU 6 m	-0.34	0.17	-0.45**	0.15
IBQ × Types/minute 6 m	-0.04	0.13	0.09	0.13	IBQ × MLU 6 m	-0.16	0.14	0.03	0.12
Mother types/minute 9 m	0.03	0.18	-0.08	0.17	Mother MLU 9 m	0.25	0.16	0.31	0.16
Mother types/minute 12 m	0.19	0.18	0.21	0.17	Mother MLU 12 m	0.39*	0.16	0.38*	0.15
Gender	0.31	0.26	0.77**	0.25	Gender	0.35	0.24	0.71**	0.23
IBQ distress to limitations	0.27	0.14	0.13	0.13	IBQ distress to limitations	0.33*	0.14	0.16	0.12
Mother types/minute 9 m	0.02	0.18	-0.05	0.17	Mother MLU 9 m	0.27	0.17	0.30	0.16
IBQ × Types/minute 9 m	-0.09	0.16	0.09	0.15	IBQ × MLU 9 m	-0.01	0.12	0.10	0.11
Mother types/minute 6 m	0.19	0.18	0.20	0.17	Mother MLU 6 m	-0.38	0.16	-0.44**	0.15
Mother types/minute 12 m	0.02	0.17	-0.04	0.16	Mother MLU 12 m	0.39	0.17	0.36*	0.15
Gender	0.31	0.26	0.77**	0.25	Gender	0.37	0.24	0.73**	0.23
IBQ distress to limitations	0.28	0.14	0.10	0.13	IBQ distress to limitations	0.35*	0.13	0.16	0.11
Mother types/minute 12 m	0.20	0.18	0.23	0.18	Mother MLU 12 m	0.49**	0.16	0.50**	0.16
IBQ × Types/minute 12 m	0.02	0.13	0.07	0.13	IBQ × MLU 12 m	0.24	0.14	0.22	0.13
Mother types/minute 6 m	0.01	0.17	-0.03	0.16	Mother MLU 6 m	-0.40*	0.17	-0.48**	0.15
Mother types/minute 9 m	0.01	0.18	-0.08	0.18	Mother MLU 9 m	0.18	0.16	0.23	0.16
Gender	0.31	0.27	0.76**	0.25	Gender	0.28	0.25	0.62**	0.23

m, months. ^∗^p < 0.05, ^∗∗^p < 0.01.

None of the models examining the moderating role of maternal lexical variability between IBQ distress to limitations and child MLU at 24 months were significant (see **Table 4**).

#### Moderator: Maternal Syntactic Complexity (i.e., MLU)

None of the moderating effects of maternal syntactic complexity between IBQ distress to limitations and child CDI at 18 months were significant, however, a significant main effect of IBQ distress to limitations was observed in the 6 months (β = 0.29, *p* = 0.04), 9 months (β = 0.33, *p* = 0.02), and 12 months (β = 0.35, *p* = 0.01) moderation models. High IBQ distress to limitations was associated with better language production at 18 months independently from maternal syntactic complexity at 6, 9, and 12 months. Despite no significant moderation effect of maternal syntactic complexity, maternal MLU demonstrated a significant main effect at 6 months (β = -0.45, *p* < 0.01) and 12 months (β = 0.50, *p* < 0.01) on 24 months child MLU production. There was no main effect of maternal MLU on 24 months child MLU in 9 months moderation models (β = 0.30, *p* = 0.80).

## Discussion

The main aim of the current study was to examine whether the quality of maternal linguistic input (i.e., maternal lexical variability and maternal syntactic complexity) moderated the association between infant temperamental characteristics (duration of orienting; smile and laughter; distress to limitations scales) and infant linguistic competence (i.e., vocabulary competence at 18 months and syntactic complexity at 24 months). We found that the association between infant attentional temperamental characteristics (i.e., IBQ duration of orienting) and vocabulary and syntactic competence at the end of the second year were moderated by the lexical variability and syntactic complexity of maternal input at 6 and 9 months of age. Specifically, infants with greater duration of orienting scores who experienced a mother speaking with greater lexical variability and more syntactic complexity at 6 and 9 months showed better linguistic skills at 18 and 24 months. Regarding the association of infant affective temperamental traits with later language development, the story appears to be more complex. We found no association (main effects or moderation) between the expression of positive affect and infant language development. However, contrary to expectations, we found a positive association between the temperamental characteristic of negative affect (i.e., distress to limitations) and later language development. Infants who were rated as having more negative affect (i.e., exhibited higher distress to limitations scores) showed better language production at 18 months when controlling for maternal syntactic complexity (i.e., maternal MLU).

Mother–infant interactions are characterized from the 5th month of life by a transition from face-to-face dyadic interactions to triadic interactions where objects become an increasing focus of verbal and attentional exchanges (Trevarthen and Aitken, 2001; Striano and Stahl, 2005). As the infant’s interest in the environment begins to grow, the mother follows the infant’s focus of interest, and scaffolds the infant’s interactions with the environment by labeling objects of interest and giving meaning to the focus of the interaction (Baumwell et al., 1997). This developmental interchange is foundational for language acquisition because infants start to detect words and to connect sounds to the referential objects and actions. The ability of the infant to pay attention to these social exchanges likely underlies the infant’s ability to learn language, and relatedly, joint attention abilities during the first year of life have been shown to facilitate language acquisition (Carpenter et al., 1998; Morales et al., 2000; Salley and Dixon, 2007). Following this rationale, we theorized that infants scoring higher on the duration of orienting scale (a measure of the infant’s ability to focus on the environment) might manifest better abilities to focus on the interactions with the mother, and consequently, to benefit from the quality of maternal input resulting in more optimal language development. Our results indicated that the infant’s attentional capacity is associated with language development, but this association is dependent on the quality of the maternal linguistic environment. In environments of medium and higher maternal lexical and syntactic complexity at 6 and 9 months, infants with higher attentional capacities (i.e., higher duration to orienting scores) manifested better language production at 18 and 24 months. Interestingly, when these highly attentive infants received a less stimulating input they showed poorer language competencies, even when compared with children with lower attentional abilities. Even for children with higher attentional capacities, language acquisition was not fostered in the absence of a stimulating linguistic environment. This could explain why some studies lack to find an association between attentional aspects and better language acquisition (c.f. Kubicek and Emde, 2012).

Surprisingly, for infants with lower attentional abilities a richer maternal input at 6 and 9 months was associated with lower language competencies. A potential explanation is that these infants tend to be more distracted during interactions and may be less able to engage in triadic interactions which include a focus on the mother and on objects. In this situation, a complex and rich linguistic environment may be an added distraction (or may be interpreted as being intrusive) for the infant, thereby contributing to suboptimal language development in less attentive infants. Consistent with this theory, Carpenter et al. (1998) found that while the quantity of maternal comments to infant’s focus of interaction at 9 and 12 months was associated with better language comprehension and production at 15 months, the quantity of lead comments typical of intrusive interaction were associated with lower language competences.

The lack of findings concerning the interaction of the temperamental trait of duration of orienting and 12-months maternal input was unexpected since previous studies found positive association’s even later (c.f. Salley and Dixon, 2007). One possible explanation is that while the complete range of joint attention skills emerge between 12 and 18 months of age (Salley and Dixon, 2007) it is possible that toward the end of the first year, individual differences in attention may cease to predict individual differences in language development thereby accounting for our non-significant moderation findings at 12-months. An alternate explanation is that the infant temperamental characteristics assessed at 3 months may not be representative of the temperamental skills at 12 months due to developmental changes over age. An additional assessment of temperament at the end of the first year could help clarify this possibility.

Contrary to what has been previously demonstrated in the literature, we found that children with more difficult temperament characterized by a greater distress to limitations at 3 months showed better language production abilities at 18 and 24 months. Our findings are supported by the branch of the literature which has suggested that negative affect expression, viewed as an emotional regulatory strategy, may create developmental opportunities by eliciting the mother’s assistance to continue engaging in goal-directed behavior (Moreno and Robinson, 2005). Greater expressions of negativity may elicit more attention from their mothers, resulting in more dyadic interchanges which may promote vocabulary acquisition. This is independent from the quality of the input they receive at 6 and 9 months. While negative affect seems to play a role in language development, we failed to find association between the expression of positive affect (e.g., higher scores on the smile and laughter scale), and language production at the end of the second year. One potential explanation for these findings is that previous research has used direct measures of positive affect expressions rather than temperamental scale derived from a questionnaire, which may contribute to our lack of a significant association. Moreover, as Laake and Bridgett (2018) reported, it is possible that previous research examining the interactions of maternal factors with infant positive affect used global measures of maternal responsiveness rather than the linguistic quality of the input, which was the focus of our study. Alternatively, our lack of findings should be interpreted with caution since our non-significant findings may be related to a small sample size which was underpowered to find significant effects of moderation.

Our study had some strengths and limitations. The longitudinal design with multiple measurements of maternal linguistic input (maternal lexical complexity and maternal syntactic complexity) and child language outcomes using both observational and parental measures is a notable strength of our study. Moreover, consistent with a transactional view of child development (Sameroff, 1975), our findings highlight the importance of both individual and contextual aspects when examining child language development.

We acknowledge some limitations of our study. First, our measurement of temperament was assessed only once, early in infancy. While this was a deliberate decision to examine the effects of early temperamental characteristics on later outcomes, it is possible that temperament could vary over age, and a replication of these findings with multiple temperamental measures would improve the strength of our findings (Dixon and Smith, 2000). Another limitation was the lack of inclusion of other dimensions of temperament to consider whether other temperamental traits are involved in the process. Another limitation is our use of parent-report measures for our assessment, rather than an observational assessment of temperamental traits. Finally, we acknowledge that we ran a number of statistical models, thus increasing the possibility of finding less reliable significance. This is the first study on the topic, and we decided to run many models in order to be able to give a complete exploratory view of the phenomena. Future studies with larger samples and other measures should aim to replicate our findings.

Despite these limitations, we believe that our findings have several implications, particularly for intervention research. When conducting interventions to improve child language abilities, temperamental aspects such as attentional control should be taken into consideration. Many intervention programs ask the mother to increase linguistic stimulation. Our findings suggest that this may be helpful if the child has good attentional abilities, while may have a detrimental effect if the child is easily distracted.

## Conclusion

The present study provides important contributions to the research on the association between temperament and language. Since development emerges in the context of bidirectional interactions between the infant and the environment it is necessary to consider the contextual factors such as the quality of maternal input when exploring the effects of temperament on language development (Sameroff, 1975). At the same time, this study highlights that temperamental characteristics contribute variably to language outcomes in different caregiving (linguistic) environments, thereby underscoring the importance of considering context when exploring the risk and protective factors in developmental science.

## Author Contributions

MS and MF developed the study concept. All authors contributed to the study design and drafted the manuscript. TA, MF, and GG performed the data collection and coding. MS performed the data analysis. MS, MF, TA, and PS wrote the final version of the manuscript and all authors approved it for submission.

## Conflict of Interest Statement

The authors declare that the research was conducted in the absence of any commercial or financial relationships that could be construed as a potential conflict of interest.

## Footnotes

1 Results and tables available upon request to authors.

## References

[B1] Aureli T. Coppola G. Picconi L. Grazia A. Ponzetti S. (2015). Relationships between regulatory temperament dimensions and self-regulatory behaviors at 4 and 6 months of age. *Infant Behav. Dev.* 38 162–166. 10.1016/j.infbeh.2014.12.013 25667170

[B2] Baer J. Schreck M. Althoff R. R. Rettew D. Harder V. Ayer L. (2015). Child temperament, maternal parenting behavior, and child social functioning. *J. Child Fam. Stud.* 24 1152–1162. 10.1007/s10826-014-9924-5 26085784 PMC4465805

[B3] Bates E. Bretherton I. Snyder L. S. (1991). *From First Words to Grammar: Individual Differences and Dissociable Mechanisms*, Vol. 20 Cambridge: Cambridge University Press.

[B4] Baumwell L. Tamis-LeMonda C. S. Bornstein M. H. (1997). Maternal verbal sensitivity and child language comprehension. *Infant Behav. Dev.* 20 247–258. 10.1016/S0163-6383(97)90026-6

[B5] Bloom L. (1990). “Developments in expression: affect and speech,” in *Psychological and Biological Approaches to Emotion*, eds Stein N. L. Bennett L. Trabasso T. (Hillsdale, NJ: Lawrence Erlbaum Associates), 215–246.

[B6] Bloom L. Beckwith R. Capatides J. B. (1988). Developments in the expression of affect. *Infant Behav. Dev.* 11 169–186. 10.1016/S0163-6383(88)80004-3

[B7] Bloom L. Capatides J. B. (1987). Expression of affect and the emergence of language. *Child Dev.* 58 1513–1522. 10.2307/1130691

[B8] Bloom L. Tinker E. Scholnick E. K. (2001). The intentionality model and language acquisition: engagement, effort, and the essential tension in development. *Monogr. Soc. Res. Child Dev.* 66 i–viii, 1–91. 11799833

[B9] Bornstein M. H. Haynes M. O. Painter K. M. (1998). Sources of child vocabulary competence: a multivariate model. *J. Child Lang.* 25 367–393. 10.1017/S0305000998003456 9770912

[B10] Canfield C. F. Saudino K. J. (2016). The influence of infant characteristics and attention to social cues on early vocabulary. *J. Exp. Child Psychol.* 150 112–129. 10.1016/j.jecp.2016.05.005 27280332

[B11] Carpenter M. Nagell K. Tomasello M. Butterworth G. Moore C. (1998). Social cognition, joint attention, and communicative competence from 9 to 15 months of age. *Monogr. Soc. Res. Child Dev.* 63 i–vi, 1–143. 10.2307/1166214 9835078

[B12] Caselli M. C. Casadio P. (1995). *Il Primo Vocabulario Del Bambino.* Milan: Franco Angeli.

[B13] Conture E. G. Kelly E. M. Walden T. A. (2013). Temperament, speech and language: an overview. *J. Commun. Disord.* 46 125–142. 10.1016/j.jcomdis.2012.11.002 23273707 PMC3630249

[B14] Coppola G. Aureli T. Grazia A. Ponzetti S. (2016). Reunion patterns in the still-face paradigm as predicted by maternal sensitivity and dyadic coordination. *Infancy* 21 453–477. 10.1111/infa.12115

[B15] Dixon W. E. Shore C. (1997). Temperamental predictors of linguistic style during multiword acquisition. *Infant Behav. Dev.* 20 99–103. 10.1016/S0163-6383(97)90065-5

[B16] Dixon W. E. Smith P. H. (2000). Links between early temperament and language acquisition. *Merrill Palmer Q.* 46 417–440.

[B17] Fasolo M. D’Odorico L. Costantini A. Cassibba R. (2010). The influence of biological, social, and developmental factors on language acquisition in pre-term born children. *Int. J. Speech Lang. Pathol.* 12 461–471. 10.3109/17549507.2011.486445 20586525

[B18] Gartstein M. A. Rothbart M. K. (2003). Studying infant temperament via the revised infant behavior questionnaire. *Infant Behav. Dev.* 26 64–86. 10.1016/S0163-6383(02)00169-8 17336002

[B19] Golinkoff R. M. Can D. D. Soderstrom M. Hirsh-Pasek K. (2015). (Baby) talk to me: the social context of infant-directed speech and its effects on early language acquisition. *Curr. Dir. Psychol. Sci.* 24 339–344. 10.1177/0963721415595345

[B20] Goodman J. C. Dale P. S. Li P. (2008). Does frequency count? Parental input and the acquisition of vocabulary. *J. Child Lang.* 35 515–531. 10.1017/S0305000907008641 18588713

[B21] Hampson J. Nelson K. (1993). The relation of maternal language to variation in rate and style of language acquisition. *J. Child Lang.* 20 313–342. 10.1017/S0305000900008308 8376472

[B22] Hayes A. F. (2017). *Introduction to Mediation, Moderation, and Conditional Process Analysis: A Regression-Based Approach.* New York, NY: Guilford Publications.

[B23] Huttenlocher J. Haight W. Bryk A. Seltzer M. Lyons T. (1991). Early vocabulary growth: relation to language input and gender. *Dev. Psychol.* 27 236–248. 10.1037/0012-1649.27.2.236 20719872

[B24] Karrass J. Braungart-Rieker J. M. (2003). Parenting and temperament as interacting agents in early language development. *Parent. Sci. Pract.* 3 235–259. 10.1207/S15327922PAR0303_03

[B25] Kitamura C. Burnham D. (2003). Pitch and communicative intent in mother’s speech: adjustments for age and sex in the first year. *Infancy* 4 85–110. 10.1207/S15327078IN0401_5

[B26] Kubicek L. F. Emde R. N. (2012). Emotional expression and language: a longitudinal study of typically developing earlier and later talkers from 15 to 30 months. *Infant Ment. Health J.* 33 553–584. 10.1002/imhj.21364 28520119

[B27] Laake L. M. Bridgett D. J. (2014). Happy babies, chatty toddlers: infant positive affect facilitates early expressive, but not receptive language. *Infant Behav. Dev.* 37 29–32. 10.1016/j.infbeh.2013.12.006 24441013 PMC4267686

[B28] Laake L. M. Bridgett D. J. (2018). Early language development in context: interactions between infant temperament and parenting characteristics. *Early Educ. Dev.* 29 730–746. 10.1080/10409289.2018.1436366

[B29] Lieven E. V. (1997). “Variation in a crosslinguistic context,” in *The Cross-Linguistic Study of Language Acquisition: Expanding the Contexts* Vol. 5 ed. Slobin D. I. (Hillsdale, NJ: Lawrence Erlbaum), 199–263.

[B30] MacWhinney B. (2000). *The CHILDES Project: Tools for Analyzing Talk: Volume I: Transcription Format and Programs, Volume II: The Database.* Cambridge, MA: MIT Press.

[B31] McNally S. Quigley J. (2014). An Irish cohort study of risk and protective factors for infant language development at 9 months. *Infant Child Dev.* 23 634–649. 10.1002/icd.1861

[B32] Molfese V. J. Rudasill K. M. Beswick J. L. Jacobi-Vessels J. L. Ferguson M. C. White J. M. (2010). Infant temperament, maternal personality, and parenting stress as contributors to infant developmental outcomes. *Merrill Palmer Q.* 56 49–79. 10.1353/mpq.0.0039

[B33] Morales M. Mundy P. Delgado C. E. Yale M. Neal R. Schwartz H. K. (2000). Gaze following, temperament, and language development in 6-month-olds: a replication and extension. *Infant Behav. Dev.* 23 231–236. 10.1016/S0163-6383(01)00038-8

[B34] Moreno A. J. Robinson J. L. (2005). Emotional vitality in infancy as a predictor of cognitive and language abilities in toddlerhood. *Infant Child Dev.* 14 383–402. 10.1002/icd.406

[B35] Newman R. S. Rowe M. L. Ratner N. B. (2016). Input and uptake at 7 months predicts toddler vocabulary: the role of child-directed speech and infant processing skills in language development. *J. Child Lang.* 43 1158–1173. 10.1017/S0305000915000446 26300377

[B36] Penela E. C. Walker O. L. Degnan K. A. Fox N. A. Henderson H. A. (2015). Early behavioral inhibition and emotion regulation: pathways toward social competence in middle childhood. *Child Dev.* 86 1227–1240. 10.1111/cdev.12384 26014351 PMC4659766

[B37] Pérez-Pereira M. Fernández P. Resches M. Gómez-Taibo M. L. (2016). Does temperament influence language development? Evidence from preterm and full-term children. *Infant Behav. Dev.* 42 11–21. 26615329 10.1016/j.infbeh.2015.10.003

[B38] Rothbart M. K. (1981). Development of individual differences in temperament. *Adv. Dev. Psychol.* 1 37–86.

[B39] Rothbart M. K. (2007). Temperament, development, and personality. *Curr. Dir. Psychol. Sci.* 16 207–212. 10.1111/j.1467-8721.2007.00505.x

[B40] Rowe M. L. (2008). Child-directed speech: relation to socioeconomic status, knowledge of child development and child vocabulary skill. *J. Child Lang.* 35 185–205. 10.1017/S0305000907008343 18300434

[B41] Rubin D. H. Crehan E. T. Althoff R. R. Rettew D. C. Krist E. Harder V. (2017). Temperamental characteristics of withdrawn behavior problems in children. *Child Psychiatry Hum. Dev.* 48 478–484. 10.1007/s10578-016-0674-z 27456111

[B42] Saffran J. R. Werker J. F. Werner L. A. (2006). “The infant’s auditory world: hearing, speech, and the beginnings of language,” in *Handbook of Child Psychology*, 6th Edn, eds Damon W. Lerner R. M. (New York, NY: John Wiley & Sons), 58–108.

[B43] Saint-Georges C. Chetouani M. Cassel R. Apicella F. Mahdhaoui A. Muratori F. (2013). Motherese in interaction: at the cross-road of emotion and cognition? (A systematic review). *PLoS One* 8:e78103. 10.1371/journal.pone.0078103 24205112 PMC3800080

[B44] Salley B. Panneton R. K. Colombo J. (2013). Separable attentional predictors of language outcome. *Infancy* 18 462–489. 10.1111/j.1532-7078.2012.00138.x 25342932 PMC4204017

[B45] Salley B. J. Dixon W. E. (2007). Temperamental and joint attentional predictors of language development. *Merrill Palmer Q.* 53 131–154. 10.1353/mpq.2007.0004 18080005 PMC2137170

[B46] Sameroff A. (1975). Transactional models in early social relations. *Hum. Dev.* 18 65–79. 10.1159/000271476

[B47] Slomkowski C. L. Nelson K. Dunn J. Plomin R. (1992). Temperament and language: relations from toddlerhood to middle childhood. *Dev. Psychol.* 28 1090–1095. 10.1037/0012-1649.28.6.1090

[B48] Soderstrom M. (2007). Beyond babytalk: re-evaluating the nature and content of speech input to preverbal infants. *Dev. Rev.* 27 501–532. 10.1016/j.dr.2007.06.002

[B49] Striano T. Stahl D. (2005). Sensitivity to triadic attention in early infancy. *Dev. Sci.* 8 333–343. 10.1111/j.1467-7687.2005.00421.x 15985067

[B50] Trevarthen C. Aitken K. J. (2001). Infant intersubjectivity: research, theory, and clinical applications. *J. Child Psychol. Psychiatry* 42 3–48. 11205623

[B51] Ullsperger J. M. Nigg J. T. Nikolas M. A. (2016). Does child temperament play a role in the association between parenting practices and child attention deficit/hyperactivity disorder? *J. Abnorm. Child Psychol.* 44 167–178. 10.1007/s10802-015-9982-1 25684446 PMC4539284

[B52] Vihman M. M. McCune L. (1994). When is a word a word? *J. Child Lang.* 21 517–542. 10.1017/S0305000900009442 7852471

[B53] Weisleder A. Fernald A. (2013). Talking to children matters: early language experience strengthens processing and builds vocabulary. *Psychol. Sci.* 24 2143–2152. 10.1177/0956797613488145 24022649 PMC5510534

[B54] Westerlund M. Lagerberg D. (2008). Expressive vocabulary in 18-month-old children in relation to demographic factors, mother and child characteristics, communication style and shared reading. *Child Care Health Dev.* 34 257–266. 10.1111/j.1365-2214.2007.00801.x 18257795

[B55] Wolfe C. D. Bell M. A. (2007). The integration of cognition and emotion during infancy and early childhood: regulatory processes associated with the development of working memory. *Brain Cogn.* 65 3–13. 10.1016/j.bandc.2006.01.009 17630061

[B56] Zampini L. Fasolo M. D’Odorico L. (2012). Characteristics of maternal input to children with down syndrome: a comparison with vocabulary size and chronological age matched groups. *First Lang.* 32 324–342. 10.1177/0142723711410780

